# Effect of Soil Type and Application of Ecological Fertilizer Composed of Ash from Biomass Combustion on Selected Physicochemical, Thermal, and Rheological Properties of Potato Starch

**DOI:** 10.3390/molecules27134318

**Published:** 2022-07-05

**Authors:** Karolina Pycia, Ewa Szupnar-Krok, Małgorzata Szostek, Renata Pawlak, Lesław Juszczak

**Affiliations:** 1 Department Food Technology and Human Nutrition, Institute of Food Technology, College of Natural Science, University of Rzeszow, Zelwerowicza 4 St., 35-601 Rzeszow, Poland; 2 Department of Crop Production, Institute of Agricultural Sciences, and Environmental Protection, College of Natural Science, University of Rzeszow, Zelwerowicza 4 St., 35-601 Rzeszow, Poland; eszpunar@ur.edu.pl (E.S.-K.); pawlak_renata@o2.pl (R.P.); 3 Department of Soil Science, Environmental Chemistry and Hydrology, University of Rzeszow, Zelwerowicza 8b St., 35-601 Rzeszow, Poland; mszostek@ur.edu.pl; 4 Department of Food Analysis and Evaluation of Food Quality, Faculty of Food Technology, University of Agriculture in Krakow, Balicka 122, 30-149 Krakow, Poland; rrjuszcz@cyf-kr.edu.pl

**Keywords:** potato, ash biomass, fertilization, starch, thermal properties, rheological properties

## Abstract

The aim of the study was to assess the effect of soil type and the application of fertilizer composed of ashes from biomass combustion to potatoes on selected physicochemical, rheological, and thermal properties of potato starches isolated by using the laboratory method. Potatoes were grown in Haplic Luvisol (HL) and Gleyic Chernozem (GC) soil and fertilized with different doses of biomass combustion ash (D1–D6) with different mineral contents. The thermodynamic characteristics of gelatinization and retrogradation were identified by DSC. The analyses of rheological properties included the determination of the gelatinization characteristics by using the RVA method, flow curves, and assessment of the viscoelastic properties of starch gels. It was found that the starches tested contained from 24.7 to 29.7 g/100 g d.m. amylose, and the clarity of 1% starch pastes ranged from 59% to 68%. The gelatinization characteristics that were determined showed statistically significant differences between the starches analyzed in terms of the tested factors. The value of maximum viscosity and final viscosity varied, respectively, in the range of 2017–2404 mPa·s and 2811–3112 mPa·s, respectively. The samples of the potato starches studied showed a non-Newtonian flow, shear thinning, and the phenomenon of thixotropy. After cooling, the starch gels showed different viscoelastic properties, all of which were weak gels (tan δ = *G*″/*G*′ > 0.1).

## 1. Introduction

Potatoes (*Solanum tuberosum* L.) are one of the most important consumer crops in the world. The annual global production of potato tubers exceeds 380 million tons [1]. Potatoes are a key raw material in various industrial processes and are also a bio-renewable source of starch with optimal functional properties [1,2]. The yield and quality of potatoes depends on many factors, the most important of which are the variety, environmental conditions, cultivation method, and type of fertilizer used. The type and size of the applied natural and artificial fertilizers applied have a significant impact on the quality and size of the yield. This is because potatoes take up large amounts of nutrients—particularly nitrogen, phosphorus, and potassium—from the soil during the growing season [1]. Thus, the quality of potatoes depends on the supply of these ingredients. The main carbohydrate component of tubers is starch, which, compared to wheat, corn, or rice starch, has a lower gelatinization temperature, higher viscosity, and clarity of the pastes [2]. These features mean that it can readily be used in food and non-food industries, both in its native and modified form. The physicochemical and rheological properties of potato starch are a function of many factors, such as the variety of potato starch, the type and method of fertilization of the crop, and the temperature and supply of water during the growing season [2]. Among the mineral elements supplied, apart from nitrogen and phosphorus, potassium is extremely important for the proper quality of the potato yield. The demand for potassium for the production of 1 ton of potatoes is approximately 4.4 kg. Potassium is also a mineral that determines the physicochemical and rheological properties of starch. Studies have shown that the fertilizing of rice with potassium decreased the amylose content in starch and, thus, facilitated its cooking [2,3]. On the other hand, other studies showed an increase in amylopectin levels in wheat starch as a result of fertilizing with potassium [2]. Zhang et al. [2] analyzed the effect of fertilizing two varieties of potato grown in China with different doses of potassium in relation to selected physicochemical properties of starch. These studies showed that fertilizing with potassium had a significant impact on the physicochemical properties of potato starch, because, with the increase in potassium supply, starch showed a greater swelling capacity, had a lower pasting and retrogradation temperature, and starch paste showed resistance to shear stresses. Phosphorus is also an important element influencing the characteristics of starch. It has been shown that, in contrast to other starches, the high phosphorus content produces high viscosity of the paste on the potato starch base [4]. In general, natural or artificial fertilizers are used in the cultivation of potatoes. Meanwhile, an ecological alternative to artificial fertilizers is ash from the combustion of plant biomass, which may dominate in this respect in the future. Currently, there is an increased demand and use of wood. As a result, the amount of forest waste is also increasing. Similarly, there is a large supply of agricultural biomass such as cereal straw, sunflower husk, and willow. This type of biomass is a valuable natural source of inorganic components in various amounts and chemical forms. The ash resulting from the combustion of forest and agricultural biomass is a natural and cheap source of potassium, phosphorus, calcium, zinc, and a number of other minerals. Thus, fertilizing agricultural crops with such ash will permit the return of these valuable nutrients to the soil, reduce the doses of the chemical fertilizers used, and, thus, reduce the costs of cultivation [5]. Therefore, research issues should be expanded on the impact of fertilizing with ashes from biomass combustion, the type of soil, and the impact of these factors on selected properties of potato starch. Such information is missing in the literature. The aim of the study was to assess the impact of soil type on fertilizing with ashes from biomass combustion and the impact of these factors on selected physicochemical, thermal, and rheological properties of potato starch.

## 2. Materials and Methods

### 2.1. Research Materials

The research material was provided by starches isolated from potatoes (*Solanum tuberosum* L.) cv. Sagitta (mid-early, potato-fries type, breeder HZPC Holland B.V., The Netherlands). The potatoes came from a field experiment involving the study of the effect of fertilizing with ashes from biomass combustion on the cultivation of potatoes. The details regarding the field trial are provided in Section 2.1.1.

The starches that were analyzed were obtained from potatoes, using the laboratory method, dried at ambient temperature (±25 °C), and then grounded and sieved through a sieve with a mesh opening of 0.125 mm [6].

#### 2.1.1. Design of Field Experiment

A field experiment located in Southeastern Poland (50°3′ N 22°47′ E) was carried out in 2020. The experiment had a two-factor design with four replications and 56 plots (plot area 40.5 m^2^). The research factors were as follows. (I) Type of soil: Haplic Luvisol and Gleyic Chernozem. (II) Varied fertilizer application to the potato: Control—only N and P fertilizer; D1—NPK mineral fertilizer; and D2–D6—N and P mineral fertilizer + ash from biomass with different doses, i.e., 0.5, 1.0, 1.5, 2.0, and 2.5 t·ha^−1^, respectively.

Mineral nitrogen fertilizer (in the form of RSM^®^ 32% N (aqueous solution of urea ammonium nitrate; density, 1.32 kg/dm^−3^)), monoammonium phosphate (MAP) NH_4_H_2_PO_4_ (12% N-NH_4_ 52% P_2_O_5_ (22.7% P)), and phosphorus (in the form of Monoammonium phosphate (MAP)) were constant. In variants D2 to D6, ashes from forest biomass combustion (70%) and agricultural biomass (30%) were used as fertilizer [5]. The potatoes were planted in the third decade of April, when the soil at a depth of 10 cm reached a temperature of approximately 6–8 °C. The cast used 40 thousand plants per ha. The tuber was harvested by hand in the third decade of September.

The experiment was carried out on two types of soils: Haplic Luvisol with a Silt (Si) particle size composition and Gleyic Chernozem with a Silty Loam (SiL) particle size composition [7]. The Haplic Luvisol soil was acidic (pH = 5.15). The reaction of Gleyic Chernozem soil was slightly acidic (pH = 5.89). The Haplic Luvisol soil has a low availability of digestible forms of P (7.19 mg·100 g^−1^); a very high availability of Mg (36.4 mg·100 g^−1^); and an average availability of K (12.85 mg·100 g^−1^), Fe (845 mg·kg^−1^), Mn (147 mg·kg^−1^), Zn (5.49 mg·kg^−1^), and Cu (2.20 mg·kg^−1^). In turn, the availability in Gleyic Chernozem soil of P (2.56 mg·100 g^−1^) was low; of K (7.23 mg·100g^−1^) was very low; of Mg (13.51 mg·100 g^−1^) was high; and of Fe (1439 mg·kg^−1^), Mn (216 mg·kg^−1^), Zn (11.39 mg·kg^−1^), and Cu (5.28 mg·kg^−1^) was average [8]. In 2020, during the potato growing season, there was 549.2 mm of precipitation, and the average air temperature was 13.9 °C.

### 2.2. Methods

#### 2.2.1. Analysis of Selected Physicochemical Properties

The isolated potato starches were analyzed for selected physicochemical parameters. The dry-matter content was determined by measuring weight loss during drying at 130 °C [6]. The apparent amylose content was determined by the spectrophotometric method, according to Morrison and Laignelet [9]. Absorbance measurements were made at the wavelength λ = 630 nm, using the ZUZI 4211/50 UV/Vis spectrophotometer (DanLab, Poland). The clarity of starch pastes was also determined by the spectrophotometric method [6,10]. The starch paste (1 g/100 g) was heated at 95 °C for 30 min, with constant stirring. The transmittance of the starch paste was measured by using the UV–Vis ZUZI 4211/50 spectrophotometer (DanLab, Poland), at a wavelength of λ = 640 nm.

The starch color analysis was also performed by spectrophotometric method, using a Color i5 spectrophotometer (X-Rite, USA). The illuminant D65 was used with the measurement geometry d/8. Measurement results were recorded in the X-Rite Color Master software. The value of was determined for the L* coordinates, describing the brightness; a*, expressing the red–green color balance; and b*, expressing the blue–yellow color balance. The analysis was performed in triplicate in the 400–700 nm spectral range. On the basis of the results obtained, the whiteness index (*WI*) [11] was calculated by using the dependence:

(1) $$WI=\sqrt{(100-L{*}^{2})+a{*}^{2}+b{*}^{2}}\;$$

#### 2.2.2. Analysis of the Content of Minerals in Starches

The contents of basic macro- and microelements (Ca, Mg, K, Na, Fe, Mn, Zn, and Cu) were determined by means of the atomic absorption spectrometry technique (HITACHI Z-2000 Tokyo, Japan) after wet mineralization in 60% HNO_3_, according to method described by Szpunar-Krok et al. [5]. Phosphorus content was determined by the spectrophotometric method [12].

#### 2.2.3. Measurement of Thermal Properties Using Differential Scanning Calorimetry (DSC)

Thermodynamic gelatinization characteristics were obtained by using a DSC F204 Phoenix (Netzsch, Germany) differential scanning calorimeter. A water–starch dispersion (3:1) was heated in the DSC aluminum pan in the temperature range of 25–110 °C, with a heating rate of 10 °C/min. The empty aluminum pan was used as a reference. On the basis of the thermograms obtained, onset (T_O_), peak (T_P_), and end set (T_E_) gelatinization temperature and enthalpy (∆H_G_) of gelatinization were calculated. After cooling, the samples were stored in the refrigerator at 4 ± 1 °C for 7 days. Retrogradation was measured by reheating the sample pans containing the starches investigated in the same conditions as for gelatinization. The onset (T_O_), peak (T_P_), and endset (T_E_) temperatures and enthalpy (∆H_R_) of retrogradation were calculated. The index of retrogradation (%R) was calculated from the ratio of ∆H retrogradation to ∆H of gelatinization [10].

#### 2.2.4. Pasting Properties by Rapid Visco Analyzer (RVA)

The gelatinization characteristics of the 5% starch suspensions were performed using an RVA viscosity analyzer (Rapid Visco Analyzer, Tec Master, Perten Instruments, Sweden). Samples (all mixed at 160 rpm) were kept at 50 °C for 1 min, then heated to 95 °C at 12 °C/min, kept at 95 °C for 3 min, cooled to 50 °C at a rate of 12 °C/min, and finally kept at 50 °C for 2 min. On the basis of the viscograms, the following values were read: gelatinization temperature (PT); maximum viscosity during heating (PV); viscosity at 95 °C (HPV); final viscosity at 50 °C (FV); decrease in viscosity during heating, value = PV-HPV (BD); and viscosity increase during cooling, value = FV-HPV (SB) [6]. The determination was performed in triplicate.

#### 2.2.5. Viscosity Curves

The starch pastes prepared in the RVA analyzer were used in these experiments. The rotary viscometer Rheolab QC (Anton-Paar, Austria) with a system of coaxial cylinders (cup diameter: 27.12 mm, bob diameter: 25.00 mm) was used for the determination of viscosity curves at temperature of 50 °C in the shear rate range from 1 to 300 s^−1^. The experimental curves were described by the power law equation:

(2) $$\eta_{ap}=K\cdot {\dot{\gamma }}^{n-1}\;$$

where *η_ap_* is the apparent viscosity (Pa·s), *K* is the consistency coefficient (Pa·s^n^), $\dot{\gamma }$ is the shear stress (s^−1^), and *n* is the flow index [6].

#### 2.2.6. Sweep Frequency Test

The starch pastes prepared in the RVA analyzer were used for these experiments. The sweep frequency test at 25 °C was carried out by using Mars II rheometer (Thermo-Haake, Germany) with cone/plate geometry (cone diameter: 60 mm, angle: 1°, gap size: 0.052 mm) [6]. Measurements were performed at constant strain of 0.01, in the linear viscoelastic region, and angular frequency range 1–100 rad/s. The power law equations were used to describe the experimental curves:

(3) $$G^{\prime }=K^{\prime }\cdot \omega^{n^{\prime }}$$

(4) $$G^{\prime \prime }=K^{\prime \prime }\cdot \omega^{n\prime \prime }$$

where *G*′ is the storage modulus (Pa); *G*″ is the loss modulus (Pa); *ω* is the angular frequency (rad/s); and *K*′, *K*″, *n*′, and *n*″ are the constants.

#### 2.2.7. Statistical Analysis

All analyses were performed in triplicate. In order to check the normality of the distribution at α = 0.05, the Shapiro–Wilk test was performed. The homogeneity of variance was also checked. For the statistical evaluation of the results, the two-way ANOVA was used. The significance of differences between the means was determined by using Tukey’s post hoc test at a significance level of 0.05. Pearson’s linear correlation coefficients were also calculated between the study parameters characterizing potato starches, and their significance was assessed at the level of 0.05. All statistical calculations were performed in TIBCO Statistica 13.3 (TIBCO Software Inc., Palo Alto, CA, USA).

## 3. Results and Discussions

### 3.1. Physicochemical Properties and Color of the Starch

Table 1 presents the parameters showing the apparent amylose content in the potato starches investigated, the clarity of 1% of the pastes, and the color of the starch. In the case of amylose content, only the type of fertilizer showed a statistically significant effect on the value of this parameter in the two-factor analysis of variance. The amylose content ranged from 24.7% (D6GC) to 29.7% (Control HL), and the average amylose content in potato starch from potatoes grown on Haplic Luvisol soil was 1.1% higher than that of those grown on the Gleyic Chernozem soil. The difference was not statistically significant. However, it was found that the lowest amylose content was found in starch from potatoes fertilized with the D6 level, i.e., with the highest potassium content. The results obtained are similar to the level of amylose content determined in potato starch by other authors [1,2,6]. Moreover, Lizarazo et al. [13] showed that the content of the linear starch fraction and its proportion in relation to amylopectin significantly depended on the cultivar of potato. The content of amylose in starch and the ratio of amylose to amylopectin determine its rheological properties, such as the viscosity of the paste, susceptibility of starch to the retrogradation process, and susceptibility to enzymatic hydrolysis [13]. Moreover, Singh et al. [14] noticed that the content of amylose in starches of various botanical origins depends on the agrotechnical conditions of the cultivation, which include the type of soil and the fertilizer applied, as well as weather conditions. Ebúrneo et al. [1] analyzed the effect of nitrogen fertilizer on the characteristics of potato starch. The cited authors showed that the content of amylose in potato starch ranged from 28.79% to 31.91%. However, no statistically significant influence of the application of nitrogen fertilizer on the value of this parameter was found. Among the mineral elements, phosphorus most strongly determines the functional and rheological properties of starch [15]. In the case of starch of botanical origin other than potato, it was found that potassium fertilizer reduced the amylose content in rice and wheat starch [3,16]. Moreover, this type of fertilizer had a positive effect on the culinary characteristics of rice [16] and the physicochemical properties of wheat starch [3]. In turn, Zhang et al. [2] found a decrease in the level of amylose content in potato starch as a result of increasing potassium levels in fertilizers used in potato cultivation. According to the authors cited, the amylose content decreased depending on the cultivar, in the range from 30.2% to 27.6% (Zhongshu 5) and from 28.1% to 26.3% (Atlantic). This may be related to the influence of potassium on the activity of enzymes involved in starch biosynthesis (SSS, SBE) [2], because this element increases the activity of these enzymes [17]. According to Zhang et al. [2], the application of potassium fertilizer reduced the activity of these enzymes and, thus, the content of amylose and phosphorus. Therefore, the research results confirm the observations of the cited authors, because, in the experiment, potatoes were fertilized with a preparation with an increasing level of potassium. The lowest amylose content was found in D6GC starch. Moreover, according to Singh and Kaur [18], the amylose content in potato starch correlates with the size of starch granules. Larger granules have an increased content of amylose and greater water absorption than smaller granules.

A high clarity of starch pastes is an important feature affecting the use of starch in the production of fruit gel, jellies, and cake fillings. The results of statistical analysis indicate that the clarity of potato-starch pastes statistically significantly depended on the type of soil, type of fertilizer used, and the interaction of these factors. The clarity of 1% starch pastes ranged from 52.5% (D3GC) to 68.0% (D5HL). The mean clarity value of starch-based pastes from potatoes grown in HL soil was 3.4% higher when compared with starch-based pastes from potatoes grown in GC soil. The fertilizer use was also an important factor in this regard, as, in general, the clarity of starch pastes depended on the chemical composition of ash from biomass combustion. Therefore, fertilizing with ash, which has the highest content of potassium (D6), resulted in an increase in clarity in tandem with an increasing share of this element. Pycia et al. [6] showed that the clarity of starch-based pastes derived from Polish potato varieties varied within a wide range (47.5–89.5%). The clarity of starch pastes depends on such factors as the concentration; the degree of starch polymerization (DP); the presence and level of non-starch components, such as proteins, fats, and minerals; and the storage period [6,19]. Moreover, Kaur et al. [20] noticed a negative correlation between the content of amylose in starch and the clarity of its paste. The clarity of pastes also changes during storage. This is related to the association of amylose chains released from starch grains as a result of the gelatinization process. This phenomenon is known as retrogradation. Zhang et al. [2] observed that, with increasing application of potassium fertilizer to potatoes, the clarity of pastes based on isolated starch increased. Therefore, according to these authors, applying potassium fertilizer to potatoes reduces the process of starch retrogradation. In our own research, a significant linear correlation was found between the clarity of starch paste and parameters such as SB, ΔH_G_, n′, and K″ (r = −0.59, r = 0.57, r = 0.60, and r = −0.50; *p* ≤ 0.05).

Color is a parameter that provides information about the quality of raw materials and the products based on them. The color of raw materials is related to their chemical composition. Changes in color parameters usually take place under the influence of storage conditions or the processing process [11]. The two-factor analysis of variance showed a statistically significant influence of fertilizer application, soil type, and interactions between these factors on the L* parameter, which is the brightness of the sample. The value of this parameter ranged from 90.2 (Control HL) to 92.9 (GCD4). The values obtained are similar to those presented by other authors [11,21]. The mean value of this parameter for starch from potatoes grown in GC was higher by 1.5 compared to HL soil. In turn, the use of D4 fertilizer in potato cultivation resulted in the formation of the highest level of brightness of the starch. The type of soil, fertilizer used, and interactions between these factors had a statistically significant effect on the value of parameter a* (in the range from green to red) and b* (in the range from blue to yellow). The b* parameter reached positive values for all the starches analyzed, which proves the color shift toward yellow. On the other hand, the values of the a* parameter in the case of HL had positive values, and in the case of GC, it had negative values, thus indicating a shift toward the green color. The mean value of the a* parameter of GC starch was 0.164, and it was five times higher than HL. Higher mean values of the b* parameter was achieved by starches from potatoes grown on HL and fertilized with D3. Whiter starch was obtained from potatoes grown in HL soil, as evidenced by the average values of the WI* parameter.

### 3.2. The Content of Mineral Elements in Starch

Table 2 presents the results relating to the mineral content of in the starches isolated from potatoes fertilized with ash from biomass combustion. The mineral composition of the ash used was dominated by potassium ranging from 99.6 kg ha^−1^year^−1^ (D1) to 469 kg ha^−1^year^−1^ (D6). However, potassium was not present in the control fertilizer [5]. The two-factor analysis of variance showed a significant statistical influence of all the factors analyzed on the phosphorus content in starch. It was found that the average content of P in starches from potatoes grown on HL soil was higher by about 5.5% compared to starches from potatoes grown on GC soil (Table 2). It was shown that the average content of phosphorus in starch generally increased with an increasing share of this element in fertilizers. The highest phosphorus content was found in potato starch fertilized with biomass ash, containing 37.7 kg·ha^−1^year^−1^ of phosphorus. The type of soil, fertilizer applied, and interactions between soil type and fertilizer used had a statistically significant influence on the level of potassium content in the studied starches (Table 2). The potassium content in starches from potatoes grown in HL was 34 mg·kg^−1^ higher than in GC soil. Control HLD6 starch had the highest content of this element, and GCD3 starch had the lowest. The two-factor analysis of variance showed the effect of soil type, fertilizer applied, and the interaction of both factors on the content of calcium, magnesium, sodium, and iron (Table 2). The Control HL starch had the highest calcium content. It was shown that, for both types of soil, there was a reduction in the Ca content of starches with the application of different doses of ash (D1–D6). Manganese and zinc were found in small amounts in the starches analyzed. The manganese content in starches was statistically significantly dependent on the type of soil and fertilizer applied. In turn, the zinc content depended solely on the ash fertilizer applied and the interaction of fertilizer applied and soil type (Table 2). The use of mineral fertilizers has a significant impact on the quality and yield of potatoes. During the growing season, potatoes take up large amounts of nutrients from the soil, especially nitrogen (N), phosphorus (P), and potassium (K) [1]. Phosphorus is an important component of potato starch, in which it mainly occurs as monoester phosphate [22]. Phosphorus, which is covalently bonded with starch granules, facilitates the incorporation of water molecules by the starch granules due to its ionic nature, thus changing its functional properties, such as gelatinization, retrogradation, and swelling power [1,15,23,24,25]. Apart from phosphorus, potato starch also has a natural content of metal cations that are attached to phosphate ester groups via ionic bonds [1,24,26,27]. Ebúrneo et al. [1] found that the application of nitrogen fertilizer to potatoes during their growth significantly influenced the size distribution of the starch grains. The authors cited found that increasing the nitrogen dose during potato cultivation resulted in an increase in the size of the starch grains and a decrease in the proportion of smaller grains in the entire spectrum of starch grains. According to Liszka-Skoczylas et al. [28], the quantitative and qualitative profile of minerals in starch may be of significant importance for its industrial application, because, for example, the degree of phosphorylation strongly determines its physicochemical and rheological properties.

### 3.3. Gelatinization Properties

Differential scanning calorimetry (DSC) makes it possible to determine the temperatures characteristic for starch gelatinization and the enthalpy of this transition. These parameters make it possible to indicate the differences between the starches isolated from potato tubers that are obtained from crops with different fertilizers applied [11]. The parameters of the thermodynamic characteristics of the gelatinization and retrogradation determined in the tests are summarized in Table 3. In the case of gelatinization, it was found that the values of the parameters To, T_P_, and T_E_ ranged from 65.2 to 66.4 °C, 70.7 to 71.2 °C, and 77.1 to 78.5 °C, respectively, for starch from potatoes grown in Haplic Luvisol soil; and they ranged from 65.9 to 67.0 °C, 70.6 to 71.8 °C, and 77.2 to 78.8 °C, respectively, for starch from potatoes grown in GC soil. The two-factor analysis of variance only showed a statistically significant influence of all test factors on the value of the transition onset temperature (T_o_). On the other hand, the value of TP was influenced by fertilizer applied and the interaction of both factors, and the TE value was only influenced by the interaction of factors. The values obtained were higher than those presented by Pycia et al. [6] or Zhang et al. [2]. The latter authors found that the value of the characteristic gelatinization temperatures of potato starch decreased with increasing potassium dose in fertilizers used in potato cultivation. According to the cited authors, the differences in gelatinization temperatures depending on the potassium dose may result from the size of starch granules and the amylose content. A higher temperature at the beginning and end of the transformation is associated with a low proportion of short chains and a high content of long-chain fragments in the starch. Moreover, it has been shown that smaller starch granules have a higher surface-to-weight ratio; therefore, they absorb water and swell more effectively than large grains, thus lowering the gelatinization temperature [10,29]. Moreover, according to Noda et al. [24], the temperature during the growing season increases the gelatinization temperature and the gelatinization enthalpy value, ΔH_G_, of potato starch. In turn, potato starch with low water absorption and water solubility has a higher value of T_o_, T_P_, and T_E_ of the gelatinization process [2]. According to Singh et al. [14], the differences in the values of transformation temperatures during starch gelatinization are significantly related to the degree of their crystallinity. A higher degree of crystallinity is associated with greater resistance of the starch to gelatinization and, thus, increases the temperature at which this process takes place. A similar correlation can be observed in the case of starch with low water absorption. Singh et al. [30] also observed a positive correlation between the degree of amylopectin polymerization and the characteristic temperatures of the gelatinization process, T_P_ and T_E_. Low temperature during the growing season of potatoes, sweet potatoes, and rice increased the gelatinization temperature and the gelatinization enthalpy value determined by the DSC method [4]. The gelatinization enthalpy, ΔH_G_, in terms of quantity and quality, provides information about the crystallinity of the starch grain and is an indicator of the loss of the granular structure of starch [14,31]. The mean value of this parameter for starch from potatoes grown in HL soil was 20.4 J/g and was 1.7 J/g higher than the value of this parameter for starch from potatoes grown in GC soil (Table 3). Zhang et al. [2] found that the ΔH values of starch from both potato cultivars tested increased with an increase of the dose of K fertilizer. According to Singh et al. [14], the gelatinization enthalpy value depends on the shape of the starch granules, the grain size distribution, and the content of bound phosphorus in the form of esters. During the thermal analysis, the values of the parameters describing the retrogradation process were also determined. When the starch paste is cooled, molecular interactions take place by creating hydrogen bonds between the starch chains. This process is called retrogradation. During this process, amylose forms bi-helical structures composed of 40–70 glucose units, while amylopectin crystallization proceeds through the formation of bonds between external short branches [6,10,14]. The values of the starch retrogradation enthalpy are usually 60–80% lower than the gelatinization enthalpy, and the transition temperatures are 10–26 °C lower than the starch retrogradation [6,10,14]. The onset temperature ranged from 46.5 °C (GCD3) to 53.0 °C (GCD2). The end of temperature (T_E_) for starches from potatoes grown on GC soil was 2.6 °C higher than on HL. The retrograde enthalpy value ranged from 5.10 (GCD2) to 8.57 J/g (HLD4). The change in the thermal properties of starch during gelatinization and during refrigerated storage depends on the ratio of amylose to amylopectin, the size and shape of the granules, and the presence of lipids. The amylose content is considered to be the main factor influencing the starch retrogradation process [23]. Based on the ratio of ΔH_R_ and ΔH_G_, the retrogradation index (R%) was calculated. The value of this parameter ranged from 29.3% (GCD3) to 44.2% (HLD4). The calculated R values are slightly lower than those presented by Pycia et al. [6] for potato starches. According to Singh et al. [14] and Pycia et al. [6], the differences in the values of the degree of retrogradation may be related to the structure of amylopectin and the degree of branch polymerization in amylopectin. Longer amylopectin chains and their greater degree of DP polymerization result in greater susceptibility of the starch polymers to retrogradation and the formation of a more crystallized structure.

### 3.4. Pasting Properties of Potato Starches

**Figure 1:**
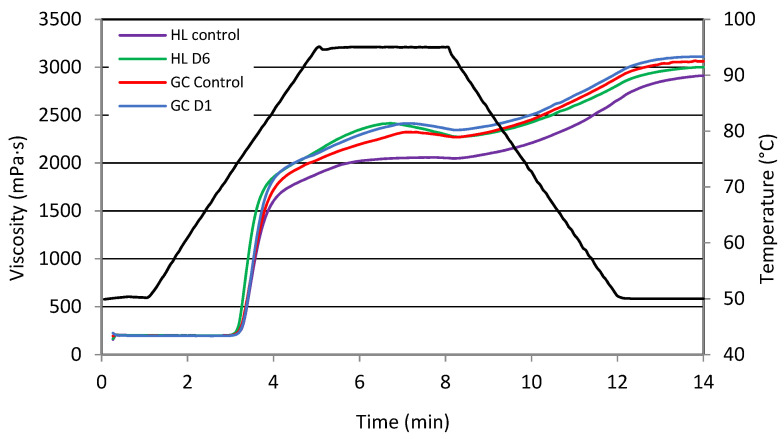
Pasting curves of selected potato starches.

When starch is heated under hydrothermal conditions, the suspension is transformed into a dispersion system, the viscosity of which changes with time and temperature. Initially, starch granules absorb water, swell, and increase in volume until they break and release amylose. The temperature at which this occurs is called the pasting temperature, and the product obtained is starch paste. The viscosity of the paste depends largely on the concentration of starch. Thus, the starch paste is a two-phase system in which entangled amylose chains are the continuous phase, and the swollen starch granules are the dispersed phase. During the cooling of such a system, an increase in viscosity is observed as a result of amylose and amylopectin chains joining hydrogen bonds [32]. Among starches of various botanical origin, potato starch is the one that has the highest viscosity, the lowest pasting temperature, and a moderate increase in viscosity during cooling. This means that potato starch easily forms paste with a high viscosity. Heating the starch suspension at a temperature above the gelatinization temperature leads to an increase in the viscosity of such a system until the maximum viscosity is reached. Further heating leads to a decrease in viscosity, and when the system is cooled, an increase in viscosity is observed. The changes described are observed in the form of the so-called pasting curves. The pasting and swelling properties are controlled in part by the molecular structure of amylopectin (unit chain length, extent of branching, molecular weight, and polydispersity), starch composition (amylose to amylopectin ratio and phosphorus content), and granule architecture (crystalline to amorphous ratio) [14,33]. Figure 1 shows examples of pasting curves of the test starches. According to Pycia et al. [6], the course of the gelatinization curves and gelatinization parameters depend on many factors, such as the plant growing conditions, size of starch granules, and presence of non-starchy substances.

The parameters of the pasting characteristics of the study starches are summarized in Table 4. A statistically significant influence was found between the type of soil and the interaction between the type of soil and fertilizer applied on the gelatinization temperature. The mean gelatinization temperature of starch from potatoes grown on HP soil was 72.3 °C and was lower by 0.3 °C compared to that from cultivation on GC soil. The fertilization of potatoes with biomass ash did not significantly affect the PT of the study starches. The parameter values that were determined are slightly higher than those reported for potato starch by other authors [2,4,6]. Zhang et al. [2] indicate that the PT value of starch influences the higher temperature and cooking time of potatoes. It was shown that the maximum PV viscosity and the HPV parameter depended on the type of fertilizer applied and the interaction of both factors tested. The value of the PV parameter ranged from 2103 mPa·s (Control HL) to 2420 mPa·s (GCD1). The effect of fertilizer application on PV was significant but inconclusive. The average PV value for starch from potatoes fertilized with D4 was 2345 mPa·s. It was shown that, on the HL soil, an increase in PV was observed with the increasing share of P and K in the fertilizer applied. The opposite was true for cultivation on GC soil. Meanwhile, Zhang et al. [2] showed that the highest PV values were achieved by potato starch at low doses of potassium. According to the authors cited, this results from the ionization of phosphate groups in the starch paste, resulting in the occurrence of Coulomb repulsion and the opening of the branched structure of amylopectin. The HPV parameter indicates the viscosity of the hot starch paste. The average HPV value for starch from potatoes grown in the HL soil was 2172 mPa·s and was lower by 27 mPa·s than that of those grown on GC soil. Continued heating of the starch paste caused a significant decrease in viscosity. The gelatinization-characteristics parameter, which determines the decrease in viscosity during further heating of the starch paste, is BD, and it had the highest values for the GCD4 and HLD6 starch pastes. It was shown that the mean BD value for starch from potatoes grown on GC soil was about 7% in relation to HL soil. The use of fertilizer increased the BD value. The highest mean BD value was recorded in starch paste based on starch from potatoes fertilized with D4. Kaur et al. [20] showed a positive correlation between PV and BD. In turn, Zhang et al. [2] found a decrease in the BD value with increasing the dose of potassium in fertilizers. Moreover, as reported by the authors cited, lower BD values indicate greater resistance of starch to heating and shear stresses. Further cooling of the paste causes an increase in viscosity shown by the SB parameter. The value of this parameter ranged from 498 mPa·s (GCD4) to 940 mPa·s (GCD2), and the type of soil in which they were cultivated did not have a statistically significantly effect on the value of this parameter. The SB value reflects the retrogradation of the starch paste during cooling, caused by the amylose leaching from the starch. Zhang et al. [2] found that SB decreased with increasing potassium dose in fertilizers. According to the authors cited, potassium fertilizer can improve the organoleptic properties of potatoes by influencing the retrogradation process, and this increases their hardness after hydrothermal treatment and during cooling. In all cases analyzed, the final viscosity FV values were higher than the maximum PV viscosity. All factors tested had a statistically significant effect on FV. The lowest value of this parameter was recorded for HLD2, and the highest was for GCD1. The statistical analysis performed showed a significant linear correlation between PV and HPV, BD, and SB (r = 0.96, r = 0.59, and r = −0.55; *p* ≤ 0.05).

### 3.5. Flow Behavior

**Figure 2:**
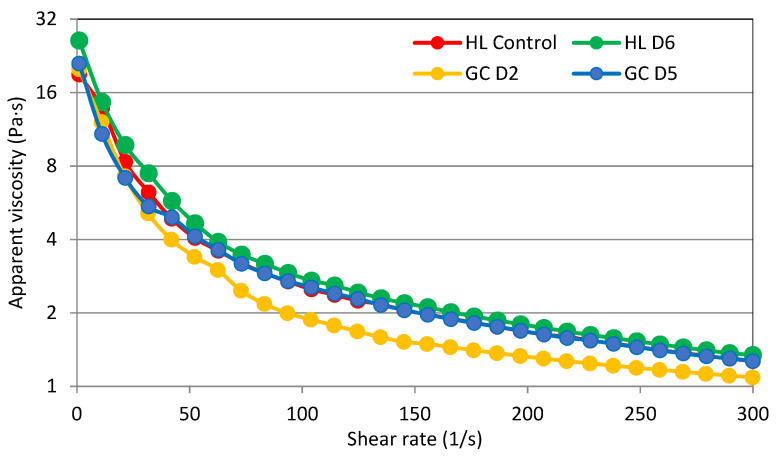
Viscosity curves of selected potato starches.

Under hydrothermal conditions, the starch suspension is transformed into a colloidal system, in which the continuous phase is formed by amylose released from starch granules, and the dispersed phase is formed by fragments of starch granules containing amylopectin. The starch paste exhibits the properties of a non-Newtonian shear-thinning fluid. The rheological properties of starch paste strongly depend on, for example, time and speed of shear, starch concentration, measurement temperature, and botanical origin of the starch [6,10]. The changes of shear stress with time and shear rate are observed in the form of curves of flow or viscosity. Viscosity curves of the potato starches analyzed are presented in Figure 2.

To describe the flow curves, a power model was used, and it described the experimental data that were obtained well. The parameters of the model used to describe the experimental curves are summarized in Table 5. The course of the viscosity curves, as well as the parameter values presented in Table 5, proves the existence of differences between the tested samples in terms of rheological properties related to viscosity. It was found that all the pastes of the tested starches showed the properties of non-Newtonian shear thinning fluids; that is, their apparent viscosity decreased with the increase of the shear rate. Based on the course of the viscosity curves, it was found that HLD6 starch had the highest apparent viscosity values in the range of shear rates applied, while GCD5 starch had the lowest values. The power model applied described the experimental curves well, as evidenced by the values of the determination coefficient, R^2^. The parameters of the power model describing the experimental curves are the coefficient of consistency (*K*) and the flow index (*n*). It was shown that all the parameters analyzed had a statistically significant influence on the value of the *K* and *n* parameter. The mean value of the consistency coefficient, which proves the initial viscosity, for starches cultivated on HL soil was about 12% higher than those obtained from cultivation on GC soil. It was shown that fertilizer application had a different effect on the *K* value. The highest K value was found for starch from potatoes fertilized with D1. The melt index ranged from 0.397 (HLD6) to 0.506 (GCD5). The melt index indicates the rate of viscosity decrease with the increase of the shear rate [6]. The statistical analysis performed showed a significant linear correlation between *K* and *n*, and the content of Mg in the starch (r = −0.81, r = 0.54; *p* ≤ 0.05), as well as between the values of the parameter n and *K*′ and *n*″ (r = −0.54, r = 0.59; *p* ≤ 0.05).

### 3.6. Viscoelastic Properties

**Figure 3:**
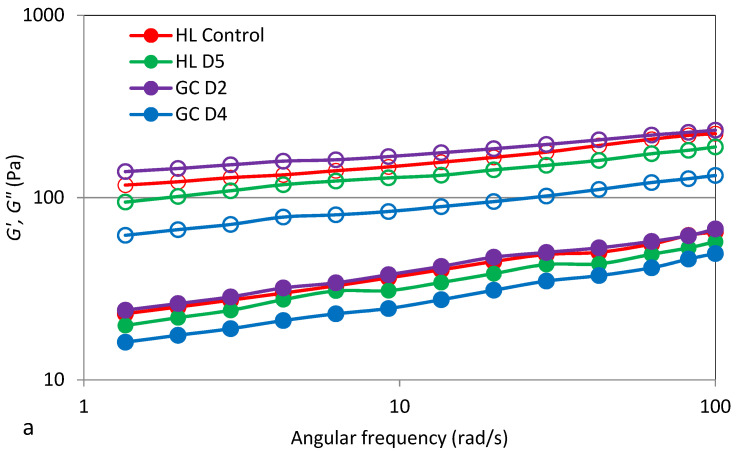

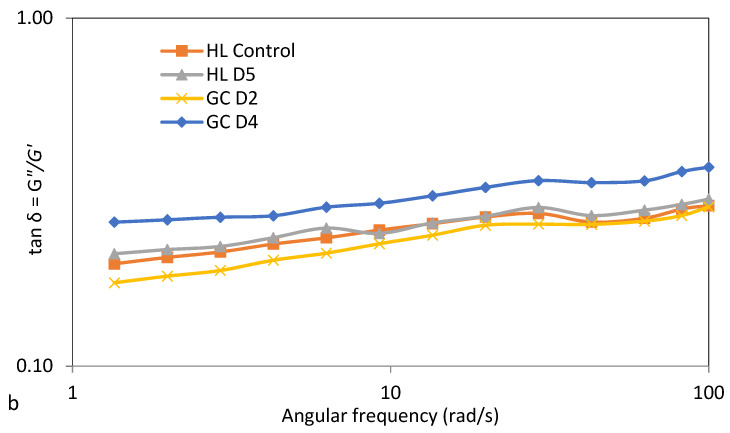
Mechanical spectra (**a**) *G*′ stands for empty markers, *G*″ filled markers, and tan δ dependence on angular frequency (**b**) of selected potato starch gels.

The use of the oscillating rheometer for rheological tests allows for the assessment of changes in the conservative modulus (*G*′) and loss (*G*″) of the starch gel, depending on the angular frequency (ω). The conservative modulus (*G*′) characterizes the share of elastic properties in the tested material and is responsible for the part of the energy that is stored. On the other hand, the loss module (*G*″) gives information about the energy lost or dispersed during deformation. The ratio of lost-to-stored energy in each cycle is described as the tangent of the phase shift angle (tan δ), which gives information about the physical behavior of the system [9,10]. Examples of mechanical spectra, i.e., the dependence of the conservative modulus (*G*′) and the loss modulus (*G*″) on the angular velocity, are shown in Figure 3a. The experimental curves determined are characteristic for weak gels, with a clear dominance of elastic properties over viscous properties and tangent values of the phase shift angle (tan δ = *G*″/*G*′) greater than 0.1 (tan > 0.1) (Figure 3b). Moreover, when comparing the values of the conservative modulus and the loss in the applied range of angular velocity plotted for all samples, it was found that, in all cases, the values of the modulus *G*′ dominated over *G*″.

The value of the *G*′ modulus depends on the amylose content in starch grains, the swelling capacity of starch grains, the degree of crystallinity, the ratio of amylose to amylopectin, the length and distribution of amylopectin chains, and the degree of amylopectin polymerization [10,34]. The experimental curves presented in Figure 3 were described by means of power equations, and the parameters obtained are summarized in Table 6. The two-factor analysis of variance showed a significant influence of the soil type, fertilizer applied, and interactions between the tested factors on the value of the constant *K*′, while the value of the constant *K*″ did not depend only on the type of soil. The value of the constant *K*′ and the constant *K*″ reflect, respectively, the modules *G*′ and G″ at an angular velocity equal to 1 rad/s. It was found that the value of the constant *K*′ representing the initial magnitude of the modulus *G*′ ranged from 92.7 (HLD5) to 131.5 (GCD2). On the other hand, the value of the constant *K*″ corresponding to the initial value of the modulus *G*″ ranged from 14.5 (GCD4) to 22.5 (GCD2). It was shown that the average highest values of the constants *K*′ and *K*″ were recorded for the cultivation of potatoes on HL soil and fertilized at the D2 level. The values of the *K*′ and *K*″ constants indicate that, in all starch gels analyzed, there was an advantage of viscous properties over elastic properties. The values of the *n*′ and *n*″ constants depended statistically significantly on the fertilizer applied and the interaction between the fertilizer applied and soil. The values of the *n*′ and *n*″ constants reflect the sensitivity of the modules to changes in angular velocity. The type of fertilizer applied and the interaction between the type of soil and fertilizer applied significantly influenced the values of these parameters. The GCD4 sample (Table 6) showed the highest value of these parameters. The statistical analysis performed showed a significant linear correlation between *K*′ and FV and SB (r = 0.69, r = 0.81, *p* ≤ 0.05), as well as the parameters *n*′, *K*″, *n*″, and FV, respectively (r = −0.61, r = 0.73, r = −0.67, *p* ≤ 0.05).

## 4. Conclusions

Ash from biomass combustion can be an ecological and valuable mineral fertilizer used for fertilizing food and starch potatoes. The research conducted proved that the minerals present in this fertilizer, to some extent, affect the physicochemical, thermal, and rheological properties of starch. Moreover, a statistically significant influence of soil type and interactions between soil and fertilizer applied on the values of most of these parameters was demonstrated. Nevertheless, the direction of these changes was not clear cut. The amylose content generally decreased as the mineral content of the starches increased. A similar tendency was observed in the case of the clarity of starch pastes, which is an important feature in terms of their use in the development of food concentrates. The highest viscosity value was characteristic for paste based on starch isolated from potatoes grown in Haplic Luvisol soil and fertilized at the D6 level. The opposite trend was observed in the case of starch derived from potatoes from Gleyic Chernozem soil. The pastes based on all tested starches showed a non-Newtonian shear-diluted flow. On the other hand, after cooling, the starch paste had the character of weak gels.

## Figures and Tables

**Table 1:** Amylose content, clarity of pastes, and color of the starch.

Type of Soil	Fertilization	Amylose Content (%)	Clarity (%)	L*	a*	b*	WI*
**Haplic Luvisol**	**Control**	29.7 ± 0.7 ^d^	59.0 ± 0.7 ^b^	90.2 ± 0.0 ^a^	0.02 ± 0.01 ^g^	2.71 ± 0.01 ^c^	10.15 ± 0.03 ^gh^
	**D1**	29.3 ± 0.8 ^d^	62.9 ± 0.2 ^cde^	90.5 ± 0.0 ^b^	−0.05 ± 0.01 ^ef^	3.00 ± 0.03 ^def^	10.00 ± 0.03 ^g^
	**D2**	27.2 ± 2.4 ^abcd^	61.0 ± 0.9 ^bc^	90.3 ± 0.1 ^ab^	0.10 ± 0.01 ^h^	2.90 ± 0.01 ^d^	10.10 ± 0.06 ^gh^
	**D3**	28.2 ± 0.9 ^bcd^	60.8 ± 20 ^bc^	91.3 ± 0.1 ^d^	−0.04 ± 0.01 ^f^	3.02 ± 0.03 ^def^	9.25 ± 0.11 ^d^
	**D4**	27.6 ± 0.9 ^abcd^	64.8 ± 1.0 ^ef^	90.9 ± 0.0 ^c^	0.02 ± 0.01 ^g^	2.91 ± 0.02 ^d^	9.52 ± 0.02 ^e^
	**D5**	25.0 ± 1.8 ^ab^	68.0 ± 1.1 ^f^	90.8 ± 0.0 ^c^	0.03 ± 0.01 ^g^	3.10 ± 0.01 ^fg^	9.72 ± 0.01 ^f^
	**D6**	26.8 ± 1.0 ^abcd^	67.1 ± 1.3 ^f^	90.2 ± 0.0 ^a^	0.10 ± 0.01 ^h^	2.97 ± 0.04 ^def^	10.26 ± 0.03 ^h^
**Gleyic Chernozem**	**Control**	25.7 ± 1.9 ^abc^	61.3 ± 0.6 ^bcd^	91.4 ± 0.0 ^de^	−0.01 ± 0.0 ^d^	3.18 ± 0.05 ^g^	9.14 ± 0.06 ^d^
	**D1**	27.5 ± 1.7 ^abcd^	59.5 ± 0.9 ^b^	92.1 ± 0.0 ^g^	−0.26 ± 0.0 ^b^	2.65 ± 0.09 ^bc^	8.35 ± 0.01 ^b^
	**D2**	28.3 ± 1.3 ^cd^	53.3 ± 0.1 ^a^	91.9 ± 0.1 ^g^	−0.12 ± 0.0 ^d^	2.54 ± 0.06 ^ab^	8.45 ± 0.10 ^b^
	**D3**	26.8 ± 0.3 ^abcd^	52.5 ± 0.3 ^a^	91.3 ± 0.0 ^d^	−0.08 ± 0.0 ^de^	3.06 ± 0.02 ^efg^	9.18 ± 0.04 ^d^
	**D4**	27.8 ± 2.1 ^abcd^	67.3 ± 1.1 ^f^	92.9 ± 0.0 ^h^	−0.30 ± 0.0 ^a^	2.48 ± 0.10 ^a^	7.54 ± 0.06 ^a^
	**D5**	25.6 ± 0.1 ^abc^	64.6 ± 0.3 ^def^	91.7 ± 0.1 ^f^	−0.2 ± 0.0 ^c^	2.96 ± 0.03 ^de^	8.81 ± 0.10 ^c^
	**D6**	24.7 ± 0.6 ^a^	61.8 ± 2.5 ^bcde^	91.6 ± 0.1 ^ef^	−0.09 ± 0.0 ^d^	2.44 ± 0.03 ^a^	8.77 ± 0.05 ^c^
**Two−Way ANOVA**							
**Type of Soil**		*p* = 0.065	*p* < 0.001	*p* < 0.001	*p* < 0.001	*p* < 0.001	*p* < 0.001
**Fertilization**		*p* < 0.001	*p* < 0.001	*p* < 0.001	*p* < 0.001	*p* < 0.001	*p* < 0.001
**Type of Soil x Fertilization**		*p* = 0.188	*p* < 0.001	*p* < 0.001	*p* < 0.001	*p* < 0.001	*p* < 0.001

Values in columns followed by the same superscript letters do not significantly differ at significance level of 0.05.

**Table 2:** The content of macronutrients and micronutrients in potato starch depending on the type of soil and application of ash fertilizer derived from biomass combustion.

Type of soil	Fertilization	P	K	Ca	Mg	Na	Fe	Mn	Zn
		**(mg⋅kg^−^^1^)**							
**Haplic** **Luvisol**	**Control**	662 ± 7 ^d^	693 ± 44 ^d^	26.9 ± 4.1 ^d^	51.5 ± 6.8 ^abcd^	6.93 ± 0.71 ^bcd^	5.29 ± 0.85 ^bcd^	0.527 ± 0.080	0.834 ± 0.184 ^ab^
	**D1**	620 ± 12 ^b^	613 ± 5 ^c^	15.7 ± 4.7 ^abc^	62.9 ± 0.8 ^e^	4.62 ± 0.42 ^ab^	5.41 ± 1.44 ^bcd^	0.899 ± 0.058	0.666 ± 0.153 ^a^
	**D2**	633 ± 24 ^bcd^	606 ± 14 ^abc^	23.4 ± 6.9 ^cd^	58.5 ± 0.3 ^de^	5.46 ± 0.05 ^abc^	5.46 ± 1.85 ^cd^	0.957 ± 0.078	0.900 ± 0.156 ^ab^
	**D3**	618 ± 12 ^b^	613 ± 21 ^c^	21.1 ± 0.5 ^bcd^	58.8 ± 3.1 ^de^	4.99 ± 2.35 ^ab^	2.91 ± 1.27 ^ab^	0.660 ± 0.434	0.639 ± 0.258 ^a^
	**D4**	630 ± 20 ^bcd^	611 ± 10 ^c^	15.4 ± 3.6 ^abc^	55.6 ± 1.4 ^cde^	5.35 ± 0.61 ^abc^	4.07 ± 0.47 ^abcd^	0.626 ± 0.059	0.716 ± 0.025 ^ab^
	**D5**	630 ± 14 ^bc^	627 ± 21 ^c^	11.0 ± 1.2 ^a^	57.0 ± 1.3 ^de^	5.04 ± 0.70 ^ab^	3.06 ± 0.52 ^acb^	0.594 ± 0.064	1.050 ± 0.062 ^ab^
	**D6**	655 ± 5 ^cd^	649 ± 13 ^cd^	10.8 ± 1.1 ^a^	55.4 ± 2.6 ^cde^	4.43 ± 0.85 ^ab^	2.53 ± 0.33 ^a^	0.681 ± 0.073	0.855 ± 0.274 ^ab^
**Gleyic** **Chernozem**	**Control**	574 ± 4 ^a^	557 ± 10 ^ab^	16.7 ± 2.9 ^abc^	56.5 ± 1.0 ^de^	2.84 ± 0.11 ^a^	3.14 ± 0.31 ^abc^	0.575 ± 0.108	1.204 ± 0.257 ^b^
	**D1**	621 ± 11 ^b^	613 ± 13 ^c^	16.9 ± 3.3 ^abc^	53.4 ± 1.5 ^abcd^	7.78 ± 0.65 ^cd^	2.97 ± 0.22 ^acb^	0.602 ± 0.115	1.020 ± 0.150 ^ab^
	**D2**	635 ± 8 ^bcd^	634 ± 14 ^c^	16.6 ± 2.0 ^abc^	48.1 ± 0.6 ^abc^	7.95 ± 0.38 ^cd^	2.13 ± 0.31 ^a^	0.561 ± 0.104	0.927 ± 0.093 ^ab^
	**D3**	564 ± 10 ^a^	553 ± 18 ^a^	11.2 ± 2.2 ^ab^	51.6 ± 2.7 ^abc^	5.56 ± 1.46 ^abc^	2.62 ± 0.65 ^a^	0.503 ± 0.051	0.581 ± 0.079 ^a^
	**D4**	579 ± 5 ^a^	601 ± 7 ^abc^	13.8 ± 3.1 ^abc^	45.8 ± 0.3 ^a^	6.51 ± 0.67 ^bcd^	2.08 ± 0.34 ^a^	0.454 ± 0.044	0.686 ± 0.132 ^a^
	**D5**	585 ± 34 ^a^	609 ± 19 ^bc^	12.2 ± 3.2 ^ab^	47.0 ± 1.7 ^ab^	4.39 ± 0.15 ^ab^	3.14 ± 0.33 ^abc^	0.428 ± 0.037	0.868 ± 0.179 ^ab^
	**D6**	644 ± 30 ^bcd^	608 ± 5 ^bc^	19.4 ± 1.8 ^abcd^	55.0 ± 0.9 ^bcde^	8.52 ± 0.36 ^d^	6.12 ± 0.76 ^d^	0.552 ± 0.138	0.673 ± 0.090 ^a^
**Two-Way ANOVA**									
**Type of Soil**		*p* < 0.001	*p* < 0.001	*p* < 0.001	*p* < 0.001	*p* < 0.001	*p* < 0.001	*p* < 0.001	*p* = 0.413
**Fertilization**		*p* < 0.001	*p* < 0.001	*p* < 0.001	*p* < 0.001	*p* < 0.001	*p* < 0.001	*p* < 0.001	*p* < 0.001
**Type of Soil x Fertilization**		*p* < 0.001	*p* < 0.001	*p* < 0.001	*p* < 0.001	*p* < 0.001	*p* < 0.001	*p* = 0.225	*p* < 0.001

Values in columns followed by the same superscript letters do not significantly differ at significance level of 0.05.

**Table 3:** Thermodynamic characteristics of gelatinization and retrogradation of potato starches.

Type of Soil	Fertilization	Gelatinization					Retrogradation					
		T_O_ (°C)	T_P_ (°C)	T_E_ (°C)	ΔT (°C)	ΔH_G_ (J/g)	T_O_ (°C)	T_P_ (°C)	T_E_ (°C)	ΔT (°C)	ΔH_R_ (J/g)	R (%)
**Haplic Luvisol**	**Control**	65.8 ± 0.5 ^ab^	71.3 ± 0.1 ^cd^	78.5 ± 1.3 ^bc^	12.7 ± 1.8 ^b^	20.3 ± 0.8 ^def^	49.5 ± 0.5 ^cd^	60.9 ± 0.6 ^a^	71.5 ± 1.4 ^a^	22.0 ± 1.6 ^ab^	6.26 ± 0.28 ^cde^	30.8 ± 1.5 ^a^
	**D1**	66.4 ± 0.3 ^bcd^	71.3 ± 0.1 ^d^	77.7 ± 0.4 ^ab^	11.3 ± 0.7 ^ab^	19.3 ± 0.8 ^cde^	50.0 ± 0.5 ^cde^	62.2 ± 0.4 ^ab^	73.9 ± 1.5 ^ab^	23.8 ± 1.6 ^b^	7.80 ± 0.33 ^h^	40.4 ± 2.6 ^e^
	**D2**	65.2 ± 0.3 ^a^	70.9 ± 0.1 ^ab^	77.4 ± 0.4 ^a^	12.2 ± 0.6 ^ab^	21.0 ± 0.3 ^ef^	50.1 ± 0.1 ^cdef^	62.2 ± 0.6 ^ab^	73.5 ± 0.7 ^ab^	23.4 ± 0.7 ^b^	7.53 ± 0.36 ^fgh^	36.0 ± 1.9 ^c^
	**D3**	66.0 ± 0.2 ^abc^	70.7 ± 0.1 ^a^	77.2 ± 0.1 ^a^	11.2 ± 0.3 ^a^	20.8 ± 0.2 ^ef^	50.4 ± 0.1 ^cdef^	62.0 ± 0.3 ^ab^	72.6 ± 0.1 ^a^	22.2 ± 0.1 ^ab^	6.81 ± 0.12 ^def^	32.7 ± 0.9 ^ab^
	**D4**	66.1 ± 0.2 ^bc^	71.1 ± 0.1 ^bcd^	77.6 ± 0.3 ^ab^	11.5 ± 0.5 ^ab^	19.4 ± 0.4 ^cde^	50.6 ± 0.2 ^def^	62.4 ± 0.3 ^b^	73.4 ± 0.2 ^ab^	22.8 ± 0.3 ^b^	8.57 ± 0.35 ^i^	44.2 ± 2.7 ^f^
	**D5**	66.0 ± 0.3 ^abc^	71.1 ± 0.1 ^bcd^	77.9 ± 0.1 ^abc^	11.9 ± 0.3 ^ab^	21.4 ± 0.5 ^f^	51.0 ± 0.1 ^efg^	62.6 ± 0.4 ^bc^	72.8 ± 0.6 ^a^	21.8 ± 0.6 ^ab^	7.49 ± 0.36 ^fgh^	35.0 ± 0.9 ^bc^
	**D6**	66.1 ± 0.1 ^bc^	71.3 ± 0.1 ^d^	77.4 ± 0.6 ^a^	11.3 ± 0.6 ^ab^	20.4 ± 0.4 ^def^	51.1 ± 0.1 ^efg^	62.7 ± 0.1 ^bc^	73.2 ± 0.1 ^ab^	22.1 ± 0.1 ^ab^	7.81 ± 0.17 ^h^	38.2 ± 1.2 ^cde^
**Gleyic Chernozem**	**Control**	66.3 ± 0.2 ^bcd^	71.3 ± 0.1 ^cd^	77.2 ± 0.7 ^a^	10.9 ± 0.9 ^a^	21.4 ± 0.5 ^f^	51.6 ± 0.2 ^fgh^	62.8 ± 0.2 ^bc^	73.6 ± 0.1 ^ab^	22.1 ± 0.1 ^ab^	7.82 ± 0.16 ^h^	36.5 ± 1.3 ^cd^
	**D1**	66.6 ± 0.3 ^bcd^	71.0 ± 0.1 ^bc^	78.1 ± 0.2 ^abc^	11.5 ± 0.5 ^ab^	16.3 ± 0.9 ^a^	52.4 ± 0.7 ^gh^	62.9 ± 0.3 ^bc^	73.6 ± 0.4 ^ab^	21.2 ± 0.5 ^ab^	7. 70 ± 0.14 ^gh^	47.3 ± 2.8 ^f^
	**D2**	67.0 ± 0.1 ^d^	71.7 ± 0.2 ^e^	78.2 ± 0.3 ^abc^	11.2 ± 0.1 ^a^	17.1 ± 0.8 ^b^	53.0 ± 0.4 ^h^	62.8 ± 0.2 ^bc^	71.8 ± 0.6 ^a^	18.8 ± 1.0 ^a^	5.10 ± 0.34 ^a^	29.9 ± 0.8 ^a^
	**D3**	65.9 ± 0.5 ^abc^	70.6 ± 0.2 ^a^	78.2 ± 0.8 ^abc^	12.3 ± 1.3 ^ab^	18.7 ± 0.9 ^bcd^	46.5 ± 0.5 ^a^	63.9 ± 0.6 ^b^	75.8 ± 1.7 ^bc^	29.3 ± 1.4 ^c^	5.47 ± 0.22 ^ab^	29.3 ± 2.1 ^a^
	**D4**	66.5 ± 0.1 ^bcd^	70.9 ± 0.2 ^ab^	77.8 ± 0.6 ^abc^	11.4 ± 0.6 ^ab^	19.4 ± 0.6 ^cde^	47.4 ± 1.3 ^a^	62.9 ± 1.1 ^bc^	78.1 ± 1.4 ^c^	30.7 ± 2.6 ^c^	5.86 ± 0.14 ^bc^	30.3 ± 1.7 ^a^
	**D5**	66.5 ± 0.3 ^bcd^	71.1 ± 0.1 ^bcd^	77.8 ± 0.1 ^abc^	11.3 ± 0.1 ^ab^	17.6 ± 0.5 ^abc^	47.6 ± 0.4 ^ab^	62.4 ± 0.2 ^b^	78.5 ± 0.7 ^c^	30.9 ± 1.1 ^c^	6.98 ± 0.19 ^efg^	39.7 ± 2.1 ^de^
	**D6**	66.7 ± 0.2 ^cd^	71.8 ± 0.3 ^e^	78.8 ± 0.1 ^c^	12.1 ± 0.1 ^ab^	20.3 ± 0.9 ^def^	49.0 ± 0.2 ^bc^	62.8 ± 0.4 ^bc^	77.9 ± 0.8 ^c^	28.9 ± 0.9 ^c^	6.12 ± 0.05 ^bcd^	30.2 ± 1.1 ^a^
**Two-Way ANOVA**												
**Type of Soil**		*p* < 0.001	*p* = 0.084	*p* = 0.053	*p* = 0.381	*p* < 0.001	*p* < 0.001	*p* < 0.001	*p* < 0.001	*p* < 0.001	*p* < 0.001	*p* < 0.001
**Fertilization**		*p* < 0.001	*p* < 0.001	*p* = 0.903	*p* = 0.963	*p* < 0.001	*p* < 0.001	*p* < 0.001	*p* < 0.001	*p* < 0.001	*p* < 0.001	*p* < 0.001
**Type of Soil x Fertilization**		*p* < 0.001	*p* < 0.001	*p* < 0.001	*p* = 0.029	*p* < 0.001	*p* < 0.001	*p* < 0.001	*p* < 0.001	*p* < 0.001	*p* < 0.001	*p* < 0.001

Mean values from three repetitions ± SD. T_O_—onset temperature; T_P_—peak temperature; T_E_—endset temperature; ΔH_G_—enthalpy of gelatinization; ΔH_R_—enthalpy of retrogradation; R—index of retrogradation = (ΔH_R_/ΔH_G_) × 100. Values in columns followed by the same superscript letters do not significantly differ at significance level of 0.05.

**Table 4:** Pasting characteristics of potato starches.

Type of Soil	Fertilization	PT (°C)	PV (mPa·s)	HPV (mPa·s)	BD (mPa·s)	FV (mPa·s)	SB (mPa·s)
**Haplic** **Luvisol**	**Control**	72.4 ± 0.7 ^ab^	2103 ± 107 ^ab^	2048 ± 105 ^ab^	55.3 ± 4.2 ^ab^	2911 ± 107 ^bcd^	863 ± 5 ^fg^
	**D1**	72.4 ± 0.1 ^ab^	2206 ± 26 ^bc^	2164 ± 28 ^bcd^	41.7 ± 3.1 ^a^	2990 ± 17^cdefg^	826 ± 18 ^defg^
	**D2**	72.2 ± 0.4 ^ab^	2017 ± 74 ^a^	1966 ± 66 ^a^	51.0 ± 7.9 ^ab^	2811 ± 68 ^bc^	844 ± 13 ^efg^
	**D3**	72.4 ± 0.8 ^ab^	2229 ± 50 ^cd^	2179 ± 42 ^bcd^	50.7 ± 9.9 ^ab^	2943 ± 30 ^bcde^	764 ± 44 ^bcd^
	**D4**	72.7 ± 0.4 ^ab^	2363 ± 29 ^e^	2263 ± 26 ^cde^	100.0 ± 7.2 ^d^	2967 ± 20 ^cdef^	718 ± 14 ^bc^
	**D5**	72.2 ± 0.9 ^ab^	2404 ± 27 ^e^	2308 ± 18 ^de^	96.0 ± 13.9 ^cd^	2998 ± 23 ^cdefg^	689 ± 35 ^b^
	**D6**	71.7 ± 0.0 ^a^	2419 ± 75 ^e^	2273 ± 47 ^de^	146.0 ± 30.5 ^e^	3001 ± 21 ^cdefg^	728 ± 29 ^bc^
**Gleyic** **Chernozem**	**Control**	72.2 ± 0.4 ^ab^	2328 ± 15 ^de^	2269 ± 14 ^de^	59.3 ± 4.7 ^abc^	3061 ± 18 ^efg^	792 ± 11 ^cdef^
	**D1**	72.9 ± 0.5 ^ab^	2420 ± 51 ^e^	2344 ± 43 ^e^	76.3 ± 9.9 ^abcd^	3112 ± 32 ^g^	768 ± 11 ^cde^
	**D2**	73.2 ± 0.0 ^ab^	2242 ± 38 ^cd^	2159 ± 37 ^bcd^	83.3 ± 1.5 ^bcd^	3099 ± 24 ^fg^	940 ± 16 ^h^
	**D3**	71.9 ± 0.5 ^ab^	2327 ± 77 ^de^	2256 ± 74 ^cde^	70.3 ± 21.0 ^abcd^	3046 ± 36 ^defg^	789 ± 40 ^cdef^
	**D4**	72.4 ± 0.1 ^ab^	2327 ± 101 ^de^	2156 ± 79 ^bcd^	170.3 ± 22.1 ^e^	2655 ± 43 ^a^	498 ± 36 ^a^
	**D5**	72.6 ± 0.0 ^ab^	2173 ± 58 ^bc^	2103 ± 6 8 ^abc^	70.0 ± 9.6 ^abcd^	2997 ± 60 ^cdefg^	895 ± 18 ^gh^
	**D6**	73.2 ± 0.0 ^b^	2112 ± 36 ^ab^	2065 ± 35 ^ab^	47.3 ± 7.6 ^ab^	2900 ± 38 ^bc^	835 ± 27 ^defg^
**Two-Way ANOVA**							
**Type of Soil**		*p* < 0.001	*p* = 0.167	*p* = 0.213	*p* = 0.224	*p* < 0.001	*p* = 0.135
**Fertilization**		*p* = 0.527	*p* < 0.001	*p* < 0.001	*p* < 0.001	*p* < 0.001	*p* < 0.001
**Type of Soil x Fertilization**		*p* < 0.001	*p* < 0.001	*p* < 0.001	*p* < 0.001	*p* < 0.001	*p* < 0.001

Mean values from three repetitions ± SD. PT—pasting temperature; PV—peak viscosity; HPV—hot paste viscosity; BD—breakdown (PV—HPV); FV—final viscosity; SB—setback (FV—HPV). Values in columns followed by the same superscript letters do not significantly differ at significance level of 0.05.

**Table 5:** Parameters of power law equations describing flow of potato starch pastes.

Type of Soil	Fertilization	*K*	*n*	R^2^
**Haplic Luvisol**	**Control**	34.2 ± 2.0 ^bcd^	0.439 ± 0.007 ^ab^	0.944
	**D1**	35.2 ± 1.0 ^d^	0.407 ± 0.003 ^a^	0.959
	**D2**	32.7 ± 1.0 ^bcd^	0.427 ± 0.021 ^ab^	0.945
	**D3**	41.5 ± 0.8 ^ef^	0.408 ± 0.018 ^a^	0.948
	**D4**	30.9 ± 0.5 ^b^	0.446 ± 0.025 ^ab^	0.949
	**D5**	39.8 ± 1.3 ^e^	0.429 ± 0.018 ^ab^	0.928
	**D6**	45.0 ± 1.5 ^g^	0.397 ± 0.013 ^a^	0.956
**Gleyic Chernozem**	**Control**	34.9 ± 1.2 ^cd^	0.429 ± 0.021 ^ab^	0.943
	**D1**	43.2 ± 0.7 ^fg^	0.399 ± 0.020 ^a^	0.956
	**D2**	31.7 ± 1.2 ^bc^	0.403 ± 0.033 ^a^	0.944
	**D3**	33.8 ± 1.2 ^bcd^	0.424 ± 0.014 ^ab^	0.945
	**D4**	25.8 ± 0.7 ^a^	0.470 ± 0.016 ^bc^	0.937
	**D5**	24.3 ± 0.1 ^a^	0.506 ± 0.001 ^c^	0.935
	**D6**	35.2 ± 1.2 ^d^	0.436 ± 0.004 ^ab^	0.933
**Two-Way ANOVA**				
**Type of Soil**		*p* < 0.001	*p* < 0.001	-
**Fertilization**		*p* < 0.001	*p* < 0.001	-
**Type of soil x Fertilization**		*p* < 0.001	*p* < 0.001	-

Mean values from three repetitions ± SD. Values in columns followed by the same superscript letters do not significantly differ at significance level of 0.05.

**Table 6:** Parameters of power law equations describing viscoelastic properties of potato starch pastes.

Type of Soil	Fertilization	*K*′	*n*′	R^2^	*K*″	*n*″	R^2^
**Haplic** **Luvisol**	**Control**	107.7 ± 6.5 ^cd^	0.155 ± 0.011 ^bc^	0.987	21.3 ± 1.9 ^b^	0.239 ± 0.006 ^cd^	0.992
	**D1**	103.3 ± 7.2 ^bcd^	0.143 ± 0.013 ^ab^	0.984	19.6 ± 1.3 ^b^	0.223 ± 0.003 ^abc^	0.982
	**D2**	103.1 ± 6.3 ^bcd^	0.145 ± 0.006 ^abc^	0.981	19.9 ± 1.4 ^b^	0.236 ± 0.012 ^cd^	0.981
	**D3**	100.4 ± 5.1 ^bcd^	0.155 ± 0.004 ^bc^	0.988	20.5 ± 2.4 ^b^	0.225 ± 0.008 ^abc^	0.992
	**D4**	96.6 ± 5.0 ^bcd^	0.155 ± 0.009 ^bc^	0.986	22.1 ± 1.2 ^b^	0.206 ± 0.012 ^a^	0.986
	**D5**	92.7 ± 3.4 ^b^	0.154 ± 0.014 ^bc^	0.990	18.9 ± 0.2 ^b^	0.235 ± 0.003 ^cd^	0.987
	**D6**	108.4 ± 5.3 ^d^	0.146 ± 0.010 ^abc^	0.993	20.3 ± 1.9 ^b^	0.231 ± 0.008 ^abcd^	0.991
**Gleyic** **Chernozem**	**Control**	103.6 ± 3.8 ^bcd^	0.143 ± 0.008 ^ab^	0.983	19.3 ± 1.4 ^b^	0.233 ± 0.00 8 ^bcd^	0.989
	**D1**	100.5 ± 2.7 ^bcd^	0.153 ± 0.007 ^bc^	0.994	21.3 ± 1.6 ^b^	0.208 ± 0.013 ^ab^	0.983
	**D2**	131.5 ± 4.4 ^e^	0.122 ± 0.012 ^a^	0.987	22.5 ± 1.3 ^b^	0.233 ± 0.013 ^bcd^	0.994
	**D3**	94.3 ± 3.6 ^bc^	0.144 ± 0.010 ^abc^	0.989	21.0 ± 1.1 ^b^	0.213 ± 0.004 ^abc^	0.980
	**D4**	58.8 ± 1.3 ^a^	0.170 ± 0.007 ^c^	0.988	14.5 ± 0.7 ^a^	0.257 ± 0.009 ^d^	0.995
	**D5**	95.6 ± 4.6 ^bcd^	0.160 ± 0.005 ^bc^	0.992	19.6 ± 1.1 ^b^	0.223 ± 0.007 ^abc^	0.989
	**D6**	93.8 ± 2.5 ^bc^	0.162 ± 0.003 ^bc^	0.991	18.9 ± 0.6 ^b^	0.234 ± 0.008 ^cd^	0.987
**Two-Way ANOVA**							
**Type of Soil**		*p* < 0.001	*p* = 1.0000	-	*p* = 0.078	*p* = 0.727	-
**Fertilization**		*p* < 0.001	*p* < 0.001	-	*p* < 0.001	*p* < 0.001	-
**Type of Soil x Fertilization**		*p* < 0.001	*p* < 0.001	-	*p* < 0.001	*p* < 0.001	-

Mean values from three repetitions ± SD. *K*′, *K*″, *n*′, and *n*″—power law equations’ constants. Values in columns followed by the same superscript letters do not significantly differ at significance level of 0.05.

## Data Availability

All data are included in the article.
